# Influenza and COVID‐19 vaccination in Canadian blood donors: A comparison across pre‐ and post‐pandemic periods

**DOI:** 10.1111/vox.70006

**Published:** 2025-02-27

**Authors:** Sheila F. O'Brien, Lori Osmond, Mindy Goldman, Steven J. Drews

**Affiliations:** ^1^ Epidemiology & Surveillance Canadian Blood Services Ottawa Ontario Canada; ^2^ School of Epidemiology & Public Health University of Ottawa Ottawa Ontario Canada; ^3^ Donation and Policy Studies Canadian Blood Services Ottawa Ontario Canada; ^4^ Department of Pathology & Laboratory Medicine University of Ottawa Ottawa Ontario Canada; ^5^ Microbiology Canadian Blood Services Edmonton Alberta Canada; ^6^ Department of Laboratory Medicine & Pathology, Faculty of Medicine & Dentistry University of Alberta Edmonton Alberta Canada

**Keywords:** blood donors, COVID‐19, influenza, SARS‐CoV‐2, vaccination

## Abstract

**Background and Objectives:**

Seasonal vaccinations reduce donor illness and appointment cancellations and ensure plasma products have antibodies to vaccine‐directed strains. We aimed to describe donor influenza and COVID‐19 vaccination history and compare this with the general population.

**Materials and Methods:**

Two online donor surveys were carried out in 2021 and 2024. Donors were asked about demographics, influenza (2019/2020, 2020/2021 and 2023/2024 seasons) and COVID‐19 (ever and 2023/2024 season) vaccination and reasons for vaccination choices. General population vaccination statistics were extracted from public reports. Percentages of donors receiving vaccination were calculated with 95% confidence intervals. Multiple logistic regression models were fitted with demographics as independent variables.

**Results:**

In survey 1, 4582 (30.4% response rate) donors completed a questionnaire; in survey 2, 6376 (21% response rate). More donors under age 65 received the influenza vaccine compared with the general population under age 65 (58% vs. 30% in 2019/2020, 63% vs. 28% in 2023/2024, *p* < 0.0001) and aged 65+ (81% vs. 70% in 2019/2020, 90% vs. 73% in 2023/2024, *p* < 0.0001). Fewer donors and the general population received the COVID‐19 vaccine in 2023/2024 (under 65 45% vs. 39%; 65+ 76% vs. 67%, *p* < 0.0001). Most said they were vaccinated to prevent infection and protect others.

**Conclusion:**

Seasonal vaccination rates are higher in older donors, consistent with public health recommendations. Blood donors are more likely to be vaccinated against seasonal influenza than the general population, but post‐pandemic uptake of the COVID‐19 booster vaccine was low, more similar to the general population.

**Highlights**

- There was a higher uptake of influenza vaccination among blood donors before, during and after the pandemic compared with the general population, suggesting blood donors were more receptive to public health recommendations.
- Uptake of the COVID‐19 booster vaccination post‐pandemic was low among blood donors and more similar to that in the general population. Public health messaging may need to be strengthened.
- A high seasonal vaccination rate among blood donors reduces their susceptibility to infection. This increases the likelihood that they will be healthy and able to donate during the influenza season. High seasonal influenza vaccination rates among blood donors ensure higher antibody levels against vaccine‐directed strains in donor plasma.

## INTRODUCTION

During the COVID‐19 pandemic, the risks of non‐transfusion‐transmissible infections to the blood supply were clear. Swift uptake of vaccines among blood donors was instrumental in ensuring an adequate blood supply during lockdowns and public health restrictions when healthy donors needed to be able and willing to attend blood collection sites. Most Canadians followed public health advice to be vaccinated during the pandemic. In Canada, blood donor uptake of the first dose of the COVID‐19 vaccine was similar to the general population [1], but in some countries, vaccination rates were higher in donors compared with the general population [2, 3, 4]. COVID‐19 is expected to continue to circulate for the foreseeable future, likely as a seasonal infection. As the SARS‐CoV‐2 virus continues to mutate, the World Health Organization reviews data and recommends vaccine strains for seasonal vaccination [5].

Vaccination uptake was monitored in blood donor SARS‐CoV‐2 serosurveys [6, 7] exemplifying the important role that blood donors can play in public health surveillance [8]. While there are publications on SARS‐CoV‐2‐specific donor vaccination under pandemic conditions, there is a paucity of data from less exceptional times. Routine childhood vaccination has been studied in relation to transfusion‐transmissible infections, mainly hepatitis B [9, 10, 11, 12], and limited blood donor seroprevalence data on other childhood immunization [13, 14, 15]. To our knowledge, seasonal vaccination such as for influenza has not been described in blood donors.

Influenza is generally considered to be the most likely infection for future pandemics due to the relative ease of transmission via social contact. Public health authorities recommend all Canadians receive influenza vaccination at the start of the winter season and particularly emphasize that those with chronic disease and those aged 65 and over receive the vaccine [16]. In the northern and southern hemispheres, influenza vaccines are revised every year to protect against the strains most likely to be circulating as recommended by the World Health Organization [17]. Although neither influenza nor COVID‐19 is transfusion‐transmissible, donors cannot donate while unwell and an understanding of the proportions of donors receiving seasonal vaccination is important for evaluating risks to the blood supply from future epidemics. Higher vaccination rates will result in greater protection from current strains of these viruses in plasma products. Comparison with general population statistics can lend insight into the suitability of donor data for public health monitoring. We conducted donor surveys asking about influenza and COVID‐19 vaccination history. We aimed to describe donor influenza and COVID‐19 vaccination history by age strata and to compare donor vaccination rates with general population statistics. We also aimed to compare donor vaccination before, during and after the COVID‐19 pandemic, and to investigate vaccination motivations and barriers.

## MATERIALS AND METHODS

Canadian Blood Services is responsible for collecting blood donations in all provinces in Canada except Quebec. Prior to donating blood, donors are asked screening questions to ensure they are in good health and are not at risk of transfusion‐transmissible infections. Their temperature is checked before donating to ensure that they are afebrile. Donors must be at least 17 years of age, but there is no upper age limit. There is no deferral period after receiving either the influenza vaccine or SARS‐CoV‐2 vaccine in Canada, since both are non‐replicating vaccines.

Two donor surveys were carried out in which all whole blood donors who had donated in the previous month and had supplied an email address were eligible to complete an online survey questionnaire, with two reminders sent after 1 week and 2 weeks. The questionnaire was developed by modifying a national telephone survey questionnaire, the Seasonal Influenza Vaccination Coverage Survey, for an online method of administration [18]. The questionnaire included demographic questions, vaccination for seasonal influenza and opinions about vaccination. In the first survey, donors were asked about influenza vaccination in the 2019/2020 or 2020/2021 fall/winter (October to April) season. In the second survey, donors were asked if they had the influenza vaccine or the COVID‐19 vaccine in the 2023/2024 fall/winter (October to April) season and if they had ever had the COVID‐19 vaccine. Donors were also asked about reasons for choosing to be vaccinated or not being vaccinated from a drop‐down menu. The list of reasons was the same for both influenza and COVID‐19 vaccination, except for influenza being received yearly was asked, whereas for the COVID‐19 booster shot, following public health recommendations was asked. Donors were also asked if they believed they had a history of COVID‐19 infection. Donors were invited to complete a questionnaire for the first survey between 27 April and 11 May 2021, and for the second survey between 16 May and 10 July 2024. Thus, they completed the survey shortly after the influenza/COVID‐19 infection risk season. Both surveys were approved by the Canadian Blood Services Research Ethics Board. Donors were provided with information about the study, and consent to participate was implied by completion of the questionnaire.

General population statistics were extracted from published reports of vaccination surveys carried out by the Public Health Agency of Canada, which asked about the same periods as the donor surveys [19, 20, 21, 22]. These data were published in two age groups (18–64 and 65+). Results of the first survey of COVID‐19 vaccination (2020/2021) were published with all adults combined [19].

The numbers of donors who donated whole blood in full years of 2021 and 2024 by demographic variables were extracted from the Epidemiology Donor Database.

### Analysis

The percentages of donors who were vaccinated from each of the vaccination questions were calculated by age groups of 17–29, 30–39, 40–49, 50–59, 60–69 and 70 and older. Results were weighted by age, sex and region to reflect the Canadian population (excluding Quebec and territories). Ninety‐five percent confidence intervals (CIs) were calculated using the Wald method. To compare the proportions of donors receiving the influenza vaccine by year, Chi‐square tests were performed. In order to compare donors with published general population results, donor answers to vaccination history questions were sorted into those aged under 65 and 65+ to match the published age bands, and percentages and 95% CIs were calculated for each of the vaccination questions. The difference between the donors and general population was calculated. Donors and general population were compared using one‐sample *t*‐tests. In order to assess the association between the vaccination and demographic variables, separate logistic regression models were constructed with the response to each question as the dependent variable. Univariate analysis with the dependent variables age group, region, gender and ethnicity was carried out. Then multiple logistic regression models were fitted for each question and all independent variables with stepwise removal of non‐significant variables based on the highest *p*‐value. Prevalence ratios were calculated by estimating the probability of vaccination for each category using the fitted logistic regression models and dividing these by the estimated probabilities for the reference categories. 95% CIs were calculated by bootstrap resampling.

Opinions about vaccination were analysed by grouping together agree and strongly agree to calculate the percentage who agreed and compared using the Wald test. To account for multiple comparisons, a *p*‐value of less than 0.01 was considered significant.

## RESULTS

In the first survey of 15,000 donors invited, 4582 (30.4%) participated. In the second survey of 29,657 donors invited, 6376 (21%) participated. The percentages of donors who participated in the surveys and donors in the donor base by demographic variables are shown in Table 1. Notably, there were fewer first‐time donors participating in the surveys in both years and fewer younger donors, but more older donors in the surveys.

**TABLE 1:** Demographic characteristics for whole blood donors in 2021 and 2024^a^ compared with donors who participated in the surveys in 2021 and 2024.^a^

	All whole blood donors in 2021 (*N* = 356,884), %	All respondents (*n* = 4582), %	*p*‐Value	All whole blood donors in 2024^a^ (*N* = 330,682), %	All respondents (*n* = 6376), %	*p*‐Value
Sex						
Male	47.9	46.3	0.0320	52.3	50.0	0.0003
Female	52.1	53.7		47.7	50.0	
Donor status						
First time	18.6	8.0	<0.0001	18.7	6.8	<0.0001
Repeat	81.4	92.0		81.3	93.2	
Age group						
Under 30	23.2	15.2	<0.0001	16.3	9.9	<0.0001
30–39	20.8	16.6		19.2	14.7	
40–49	17.1	16.1		18.9	15.0	
50–59	18.4	23.1		18.9	20.8	
60–69	15.6	22.3		19.0	28.1	
70+	4.9	6.8		7.8	11.5	
Region						
British Columbia	16.8	15.2	<0.0001	17.5	29.5	<0.0001
Alberta	18.3	16.1		17.7	17.5	
Prairies	10.3	9.1		9.9	9.8	
Ontario	45.1	53.8		44.7	28.6	
Atlantic	9.5	5.8		10.2	14.6	

^a^ January to September 2024.

**FIGURE 1:**
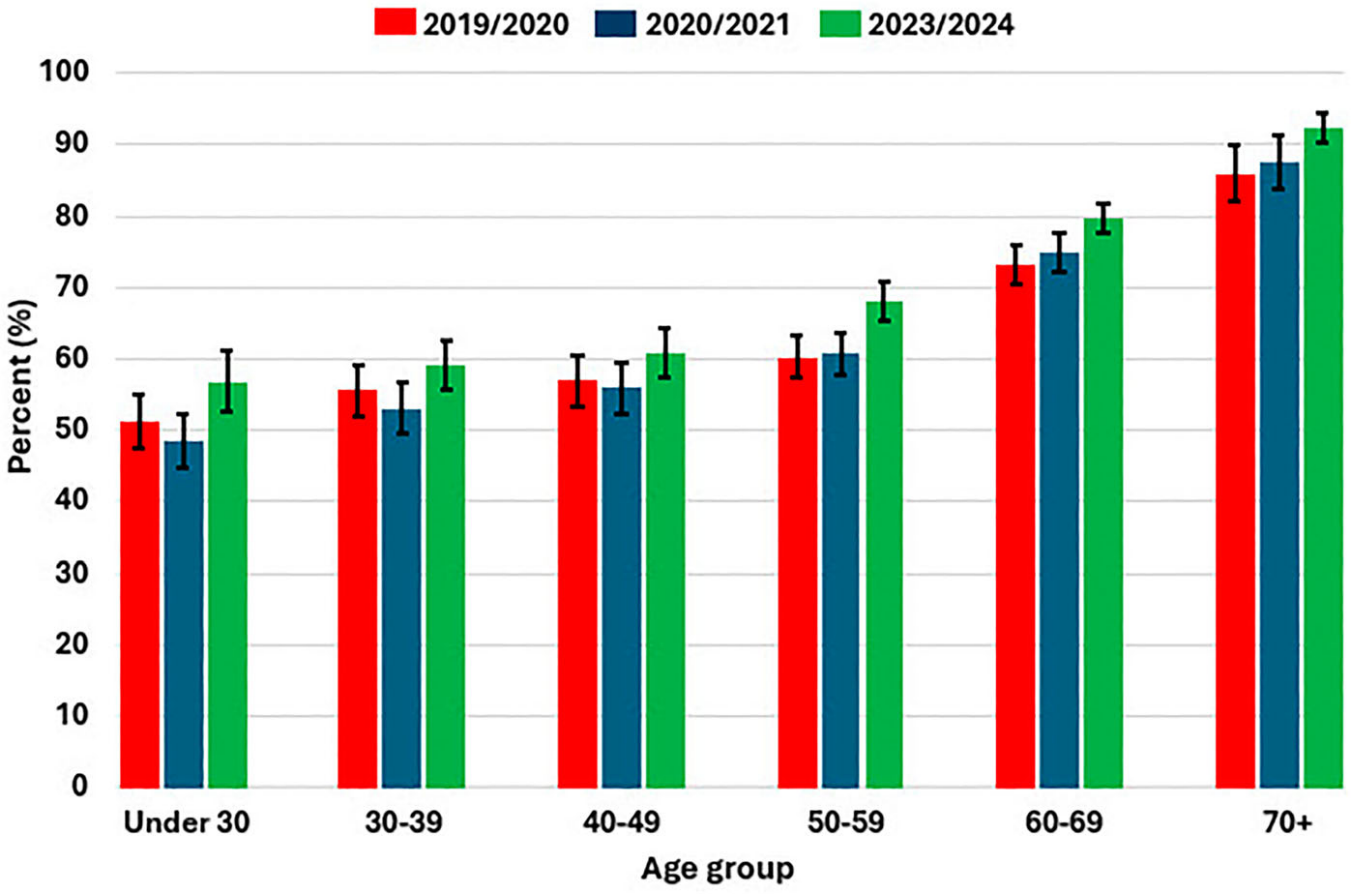
Percentage of donors who reported having influenza vaccination by age group and year.

The percentage of donors who said they received the influenza vaccine increased with age in 2019/2020 before the COVID‐19 pandemic, in 2020/2021 during the pandemic and in 2023/2024 post‐pandemic (*p* < 0.0001) (Figure 1). Nearly twice as many donors under 65 years old had influenza vaccination than the general population under 65 in any of these years, with the difference being between 28% and 35% (Table 2). Among those 65 or older, the percentage of donors who had the influenza vaccine was also higher than the general population over age 65, but by a lesser percentage, with the difference being between 11% and 17%. While the percentage of the general population who had influenza vaccination was largely unchanged over the three seasons studied, donors were somewhat more likely to be vaccinated post‐pandemic (*p* < 0.0001) (Figure 1, Table 2).

**TABLE 2:** Percentages of donors and the general population who reported having influenza vaccination and COVID‐19 vaccination by season and age group.

	Donors, 18–64	General population, 18–64	*p*‐Value	Difference, 18–64	Donors, 65+	General population, 65+	*p*‐Value	Difference, 65+
Influenza vaccinated								
2019/2020	57.8 (56.2, 59.3)	30	<0.0001	27.8	81.2 (78.5, 83.9)	70	<0.0001	11.2
2020/2021	56.6 (55.0, 58.2)	29	<0.0001	27.6	83.2 (80.0, 85.2)	70	<0.0001	13.2
2023/2024	62.9 (61.4, 64.4)	28	<0.0001	34.9	89.6 (88.0, 91.2)	73	<0.0001	16.6
COVID‐19 vaccinated								
2023/2024	44.6 (43.2, 46.0)	39	<0.0001	5.6	76.4 (74.3, 78.5)	67	<0.0001	9.4

**FIGURE 2:**
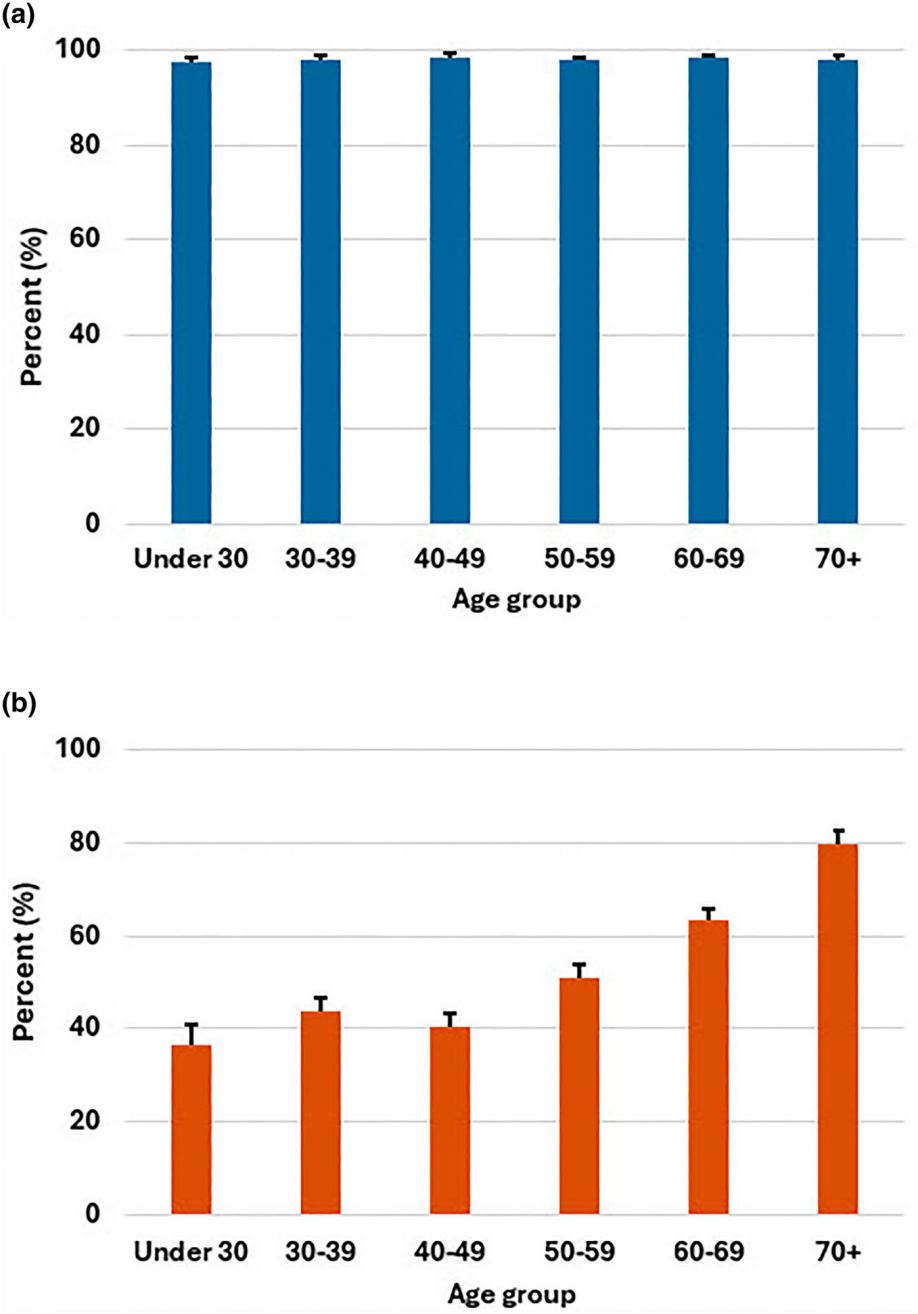
Percentage of donors by age group who reported ever having COVID‐19 vaccination (a) and who reported having COVID‐19 vaccination in the 2023/2024 season (b).

By 2023/2024 nearly all donors had been vaccinated against COVID‐19 (97.9%), but fewer chose to have their seasonal booster vaccine (Figure 2). The percentages of donors who had the 2023/2024 COVID‐19 booster vaccine were higher compared with the general population (18–64 years, 45% vs. 39% [difference 6%], 65+ years, 77% vs. 67% [difference 9%]) (Table 2). Overall, in the 2023/2024 season, 45.0% of donors had both influenza and COVID‐19 vaccinations, 13.1% had influenza vaccination but not COVID‐19, 4.4% had COVID‐19 but not influenza vaccination and 37.4% had neither.

In logistic regression for influenza vaccination, older age was consistently predictive of vaccination, but there was variability in other predictors by season (Table 3). By 2024, most donors had received at least one dose of the COVID‐19 vaccine (97.9%) and differences in odds ratios of independent variables were small (results not shown). Post‐pandemic donor uptake of the COVID‐19 vaccine was associated with older age and was lower in racialized donors.

**TABLE 3:** Output of multivariable logistic regression models with influenza vaccination in 2019/2020 (a), 2020/2021 (b), 2023/2024 (c) and COVID‐19 booster 2023/2024 (d) as the dependent variables with prevalence ratios.^a^

	(a) Influenza vaccination 2019/2020			
	95% CI			
	PR	Lower	Upper	*p*‐Value
Age group				
Under 30^a^				
30–39	1.1	1.0	1.2	0.1823
40–49	1.1	1.0	1.2	0.1731
50–59	**1.1**	**1.1**	**1.2**	**0.0055**
60–69	**1.4**	**1.3**	**1.5**	**<0.0001**
70+	**1.6**	**1.5**	**1.8**	**<0.0001**
Region				
Ontario^a^				
British Columbia	1.0	0.9	1.0	0.2849
Alberta	**1.1**	**1.1**	**1.2**	**<0.0001**
Prairies	**1.1**	**1.0**	**1.2**	**0.0240**
Atlantic	**1.2**	**1.1**	**1.3**	**<0.0001**

	(b) Influenza vaccination 2020/2021			
	95% CI			
	PR	Lower	Upper	*p*‐Value
Age group				
Under 30^a^				
30–39	1.1	1.0	1.2	0.1991
40–49	1.1	1.0	1.2	0.0846
50–59	**1.2**	**1.1**	**1.3**	**<0.0001**
60–69	**1.5**	**1.4**	**1.6**	**<0.0001**
70+	**1.7**	**1.6**	**1.8**	**<0.0001**
Sex				
Male^a^				
Female	1.1	1.0	1.1	0.0049
Region				
Ontario^a^				
BC	1.0	0.9	1.1	0.6885
Alberta	**1.2**	**1.1**	**1.2**	**<0.0001**
Prairies	**1.2**	**1.1**	**1.3**	**<0.0001**
Atlantic	**1.3**	**1.2**	**1.3**	**<0.0001**

	(c) Influenza vaccination 2023/2024			
	95% CI			
	PR	Lower	Upper	*p*‐Value
Age group				
Under 30^a^				
30–39	1.1	1.0	1.2	0.2688
40–49	**1.1**	**1.0**	**1.2**	**0.0346**
50–59	**1.2**	**1.1**	**1.3**	**<0.0001**
60–69	**1.4**	**1.3**	**1.5**	**<0.0001**
70+	**1.6**	**1.5**	**1.7**	**<0.0001**
Region				
Ontario^a^				
BC	**1.1**	**1.1**	**1.2**	**<0.0001**
Alberta	1.0	1.0	1.1	0.4082
Prairies	1.0	1.0	1.1	0.1060
Atlantic	1.0	1.0	1.1	0.1529
Other^a^				

	(d) COVID‐19 booster 2023/2024			
	95% CI			
	PR	Lower	Upper	*p*‐Value
Age group				
Under 30^a^				
30–39	**1.2**	**1.0**	**1.3**	**0.0084**
40–49	**1.1**	**1.0**	**1.3**	**0.0340**
50–59	**1.4**	**1.2**	**1.6**	**<0.0001**
60–69	**1.7**	**1.5**	**1.9**	**<0.0001**
70+	**2.1**	**1.9**	**2.3**	**<0.0001**
Region				
Ontario^a^				
BC	**1.3**	**1.2**	**1.4**	**<0.0001**
Alberta	1.0	1.0	1.1	0.4370
Prairies	**1.2**	**1.1**	**1.3**	**0.0001**
Atlantic	1.0	0.9	1.1	0.8127
Ethnicity				
White	**1.1**	**1.0**	**1.2**	**<0.0001**
Other^a^				

*Note*: Significant prevalence ratios are highlighted in bold.

Abbreviation: CI, confidence interval; PR, prevalence ratio.

^a^ Reference.

In 2023/2024, the most common reasons for choosing to have the influenza vaccination were to prevent infection (80% aged under 65, 72% 65+, *p* < 0.0001), to protect others (53% aged under 65, 38% 65+, *p* < 0.0001) and they routinely receive each year (46% aged under 65, 52% 65+, *p* = 0.0009). Of those who chose not to have the influenza vaccination, the most common reasons were a belief that they never get the flu (8% aged under 65, 16% 65+, *p* < 0.0001), not at high risk (9% aged under 65, 13% 65+, *p* = 0.07) but most said there was no specific reason (67% aged under 65, 55% 65+, *p* = 0.006). Many donors believed they had been infected with COVID‐19 in the past (76% aged under 65, 61% 65+, *p* < 0.0001). The most common reasons for having the COVID‐19 booster vaccine in 2023/2024 were to prevent infection (90% aged under 65, 88% 65+, *p* = 0.05), to protect others (83% aged under 65, 73% 65+, *p* < 0.0001) and to follow public health recommendations (71% aged under 65, 72% 65+, *p* = 0.8).

## DISCUSSION

Donors had consistently higher vaccination rates for influenza before, during and after the pandemic compared with the general population. Donors were somewhat more likely to receive the influenza vaccine post‐pandemic, whereas the general population had similar rates pre‐ and post‐pandemic. Nearly all donors and the general population had received COVID‐19 vaccination at some stage, but fewer donors and the general population chose to receive the booster shot post‐pandemic, notably fewer than those who chose to receive influenza vaccination.

In most jurisdictions in Canada, seasonal vaccination for influenza and COVID‐19 is available free of charge to residents. People may be vaccinated through general practitioners, city‐managed community and workplace pop‐up clinics, and through pharmacies. Pharmacies have been able to administer seasonal vaccines for about 10 years, with increasing uptake over the COVID‐19 pandemic. This has made vaccination more accessible to many communities.

Influenza is a common infection affecting 5%–10% of adults worldwide each year [16]. Influenza infection is often relatively mild, but it can be serious, leading to urgent care, hospitalization and death. Canadian guidelines recommend that all Canadians over 6 months of age receive influenza vaccination each year [16]. Most provinces have set targets of 80% coverage of adults aged 65 and older, adults with chronic disease and healthcare professionals. The Canadian Pediatric Society recommends that all children and youth ≥6 months of age receive an annual influenza vaccine, as well as adults who may transmit influenza to children and youth at high risk [23]. Because older adults tend to have waning protection earlier than younger people [24], adults aged 65 and older are eligible for a higher dose vaccination [16]. Public health messaging tends to focus on those aged 65 and older, younger children and younger people with chronic disease. As shown in our logistic regression analyses, older age was associated with vaccination in all three seasons studied, independent of sex, ethnicity and region.

Blood donors were more likely to have influenza vaccination at all time points studied and in both age groups. The most common reasons for being vaccinated were to not get sick and to protect others, and because they get it every year. Among blood donors aged 65 and over, the percentage of donors vaccinated in each year studied was consistently over 80%, whereas the estimated percentage of people in the general population was consistently below 80%. It may be expected that more of the general population aged 65+ were both older and living with more chronic conditions than donors yet were less likely to be vaccinated. Vaccination rates were lower in younger donors (under 65) and the general population, but still the rate in donors was about twice that of the general population. Serious illness is less frequent in younger adults, and younger people may be less concerned [25]. The higher vaccination rates we observe in donors suggest that blood donors may be more health conscious and more willing to seek preventive care [26].

COVID‐19 vaccination was first available in Canada in late December 2020, with very high uptake [27]. By the end of 2021, 94% of Canadian adults had received at least one dose, and nearly all donors had as well [1]. During the pandemic, with lockdowns and closures to prevent infection, the motivation to be vaccinated was very high. In the latter half of 2021, many jurisdictions introduced rules that barred entry to most public spaces without proof of vaccination, further emphasizing the importance of being vaccinated. Most of the restrictions were removed by late 2022. Past COVID‐19 vaccination, as well as past infection, may provide some protection with milder symptoms but do not prevent infection altogether, and symptoms can still be severe [28]. COVID‐19 vaccination is recommended for all Canadians aged 6 months or older, and particularly for those aged 65 or older and those at high risk [16]. The vaccine was revised consistent with circulating strains (Omicron XBB1.5), and in 2023/2024, vaccination reduced medically attended infections by 47%, with a higher reduction in people with a confirmed prior infection (67%) [29].

Vaccination saves lives and reduces the burden on acute healthcare. In the 2023/2024 season, Canadians who had the influenza vaccine had a 60% reduction in the risk of medically attended influenza A (H1N1) infection, a 40% reduction in influenza A (H3N2) infection [29] and a 75% reduction in influenza B [30]. Vaccination protects recipients of the vaccine but also reduces the risk of those unvaccinated by reducing exposure. Influenza infection rates were lower over the pandemic with reduced social contacts, but by 2022/2023, they had returned to seasonal epidemic levels [25]. Data from both the general population and blood donors presented in this report underscore the ongoing issue of encouraging seasonal vaccination.

Our analysis highlights important changes in vaccination rates post‐pandemic. Nearly two thirds (62.9%) of blood donors aged 18–64 had influenza vaccination in the 2023/2024 season, but less than half (44.6%) had COVID‐19 vaccination. In the general population aged 18–64, the influenza vaccination rate was lower (28%), but the COVID‐19 vaccination rate was rather similar to donors (39%). COVID‐19 vaccination rates were higher in both donors and the general population aged 65+, but COVID‐19 vaccination rates were still somewhat lower than influenza vaccination rates in this age group. Racialized donors were somewhat less likely to receive COVID‐19 vaccination, as was also seen during the pandemic, but this was not observed for the influenza vaccine [31].

For more than 20 years, free influenza vaccination has been available with regular encouragement from public health and primary care physicians. In earlier years, advertisements encouraged all Canadians to be vaccinated, not just older and high‐risk individuals. Awareness of the importance of the influenza vaccine is therefore well entrenched. COVID‐19 vaccination, on the other hand, was in its third year of being available. Amid disruption to people's lives during the pandemic, COVID‐19 risks and then vaccination had exceptionally high visibility. With the restrictions lifted and the pandemic declared over, that high visibility has abated. To some degree, past vaccination and infection lower the risk of severe COVID‐19 infection [32]. The impact of vaccine and infection‐derived immunity in influenza infection and disease may be impacted by other factors and may involve immune imprinting [33, 34]. However, circulating strains of both influenza and COVID‐19 continue to change, and vaccines are revised in response. With each new season, the extent of the epidemic to come is not fully predictable. Donors appear more likely to take heed of public health messaging with higher influenza vaccination rates than the general population. The lower rates of COVID‐19 vaccination in donors, more similar to the general population, have also been reported in the United States and suggest that public health messaging needs to be strengthened [35]. In the 2023/2024 season, flu vaccination clinics sometimes did not offer COVID‐19 vaccination, which may have decreased COVID‐19 vaccine uptake. Preparations that combine influenza and COVID‐19 vaccine could improve uptake. Monitoring donor vaccination rates may give important insight into how well public health messaging resonates, as they may respond more readily. Further studies in donor seasonal vaccination are required to better understand this.

Higher rates of vaccination in donors could be important in the event of another pandemic for several reasons. To ensure a sufficient blood supply, donors must be well and able to donate and be less susceptible to seasonal viruses that may be circulating in addition to the pandemic agent. They may be more accepting of new vaccines during a pandemic. Higher vaccination rates also mean that donor plasma will have antibodies to vaccine‐directed strains.

Our study has some important limitations. There is potential response bias with a 20%–30% response rate. The general population survey did not report denominators by age groups; hence, statistical comparison with donors was limited. All vaccination data were self‐reported.

In conclusion, blood donors are more likely to be vaccinated for seasonal influenza than the general population. Post‐pandemic COVID‐19 vaccination rates were lower in donors than influenza vaccination rates, especially in younger donors, and more similar to the general population. Public health messaging regarding the ongoing need for protection against COVID‐19 in the post‐pandemic period may need to be stronger, and other ways to encourage vaccination should be explored. Monitoring donor vaccination rates may be informative of the impact as donors may respond more readily.

## CONFLICT OF INTEREST STATEMENT

The authors declare no conflicts of interest.

## Data Availability

The data that support the findings of this study are available on request from the corresponding author. The data are not publicly available due to privacy or ethical restrictions.
